# From Swiffers to Solutions: The Impact of Environmental Sampling in the Veterinary Medical Center at OSU

**DOI:** 10.1017/ash.2024.339

**Published:** 2024-09-16

**Authors:** Christy King, Thomas Wittum, Dixie Mollenkopf, Dubraska Diaz-Campos, Joany Van Balen

**Affiliations:** The Ohio State University, College of Veterinary Medicine

## Abstract

**Background:** In 2018, the Ohio State University College of Veterinary Medicine (OSU CVM) implemented an Antimicrobial Stewardship Program, central to which was the integration of an environmental surveillance (ES) program. The ES focuses on pathogens recognized as urgent threats to public health by the Centers for Disease Control and Prevention. The pathogens currently targeted include carbapenemase-producing Enterobacterales (CPE), Salmonella spp., methicillin-resistant Staphylococcus spp. (MRSs), vancomycin resistant Enterococcus spp., and enrofloxacin resistant Pseudomonas aeruginosa. Identification of these pathogens allows the hospital to be aware of the local environmental microflora which can act as a sentinel for disease in the hospital, potentially causing healthcare associated infections. Therefore, the objective of this program is to identify resistant bacterial pathogens, characterize their resistance profiles, analyze prevalence patterns, and initiate infection control interventions where needed in the OSU VMC. **Method:** From January 2018 through December 2023, a total of 5449 samples were collected from approximately 86 locations across the OSU VMC encompassing the small animal, equine, and farm animal sections. A majority (64%, n=3561) of samples were collected from the small animal hospital, with the farm animal section contributing 1055 samples and the equine section 899. Areas sampled were frequented by both humans and animals, as well as surfaces exclusively touched by humans. Samples were collected using Swiffers® and processed through selective culture media. **Result:** Approximately half (52%, n=2890) of the samples collected represented human-touch only surfaces. A total of 3794 bacterial isolates were recovered, with an overall low prevalence for all targeted pathogens. Prevalence of CPE was 2% (n=103), with Enterobacter species being the most common. Recovery of MRSs was 8.5% (n=464) and Salmonella species was 1% (n=47). **Conclusion:** Through this initiative, the equine division of the OSU VMC collaborated with the antimicrobial stewardship team to enhance their Salmonella fecal and ES practices. In 2019, ES was critical in identifying persistent CPE and extended-spectrum cephalosporin-resistant Enterobacteriaceae in the ICU and surrounding areas of the small animal hospital. Effective measures were taken to halt the spread of ESC among patients and eliminate CPE in the environment. With the discovery of a new CPE in early 2023 in the small animal ICU and nearby areas, the program initiated targeted ES and cleaning and disinfection protocols, to identify contaminated areas and control disease transmission. These efforts have increased patient safety, health, and well-being, demonstrating how ES can be an important tool for infection control and prevention in veterinary settings.

